# *MiR-138* protects cardiac cells against hypoxia through modulation of glucose metabolism by targetting pyruvate dehydrogenase kinase 1

**DOI:** 10.1042/BSR20170296

**Published:** 2017-11-21

**Authors:** Hang Zhu, Hao Xue, Qin-Hua Jin, Jun Guo, Yun-Dai Chen

**Affiliations:** Department of Cardiology, General Hospital of Chinese PLA, Beijing 100853, China

**Keywords:** cardiac cells, hypoxia-induced apoptosis, MiR-138, Pyruvate Dehydrogenase Kinase 1

## Abstract

Dysfunction of cardiac cells under hypoxia has been identified as an essential event leading to myocytes functional failure. MiRNAs are importantly regulatory small-noncoding RNAs that negatively regulate gene expression through the direct binding of 3′-UTR region of their target mRNAs. Recent studies have demonstrated that miRNAs are aberrantly expressed in the cardiovascular system under pathological conditions.Pyruvate dehydrogenase kinase 1 (PDK1) is a kinase which phosphorylates pyruvate dehydrogenase to inactivate it, leading to elevated anaerobic glycolysis and decreased cellular respiration. In the present study, we report that *miR-138* expressions were significantly suppressed under long exposure to hypoxia. In addition, overexpression of *miR-138* protects human cardiac cells against hypoxia. We observed *miR-138* inhibits glycolysis but promotes mitochondrial respiration through directly targetting PDK1. Moreover, we demonstrate that hypoxia induces cardiac cell death through increased glycolysis and decreased mitochondrial respiration. Inhibition of glycolysis by either glycolysis inhibitor or knockdown glycolysis enzymes, Glucose transportor 1 (Glut1) or PDK1 contributes to cardiac cells’ survival. The cell sentivity to hypoxia was recovered when the PDK1 level was restored in miR-138 overexpressing cardiac cells. The present study leads to the intervention of novel therapeutic strategies against cardiac cells dysfunction during surgery or ischemia.

## Introduction

Heart failure, a heart disease caused by myocardial injury, results in high mortality [1]. Multiple factors are involved in the etiology of heart failure such as oxidative stress [2], hypoxic condition [3], stimulation by inflammation factor or cytokines [4], and injury of cardiac tissues [5]. Amongst them, apoptosis of cardiac muscle cells under hypoxia has been identified as an essential event leading to myocytes functional failure, fibrosis, and ensuing ventricular remodeling [6]. During the myocardical infraction,occusion of coronary arties deprives the oxygen of myocardium. Moreover, hypoxia may persist within the infarct for days or weeks, exacerbating the injury [7]. Therefore, understanding the molecular mechanisms of the hypoxia-induced cardiomyocytes’ death contributes to the development of therapeutic approaches for the treatment of ischemia-induced myocardial injury.

Under normal conditions, heart generates energy for fuel contractile function and viability oxidation through fatty acids oxidation [8]. However, the impaired myocardial tissues switch to other metabolic pathways by accelerated utilization of glucose, lactate, ketones, and amino acids [9]. Pyruvate dehydrogenase complex (PDC) is a complex of three enzymes that convert pyruvate into acetyl-CoA [10], which will be used in the citric acid cycle for the processes of oxidative phosphorylation [10]. Pyruvate dehydrogenase kinase (PDK) is a kinase which phosphorylates PDC to inactivate it [11]. Thus, increased PDK expression or activity will prevent the incorporation of pyruvate into oxidative phosphorylation process, leading to elevated anaerobic glycolysis and decreased cellular respiration.

MiRNAs have been recognized as an important regulatory small-noncoding RNAs that negatively regulate gene expression through the direct binding of 3′-UTR region of their target mRNAs [12]. Recent studies have demonstrated that miRNAs are aberrantly expressed in the cardiovascular system under pathological conditions and play essential roles in cardiovascular diseases including cardiomyopathy [13], cardiac fibrosis [14], cardiomyocytes dysfunction [15], cardiac ischemia [16], and arrhythmia [17]. *MiR-138* has been reported as one of the miRNAs that functions in heart diseases [18]. Interestingly, previous studies reported that *miR-138* expression was induced in hypoxic cardiomyocytes and regulated the hypoxia-induced cell apoptosis [19]. However, the mechanisms underlying that *miR-138* protects cardiomyocytes from hypoxia-induced apoptosis were not completely understood. In the present study, we will study the roles of *miR-138* in regulating cardiac cells death during hypoxia. The functions of *miR-138* in cellular glucose metabolism will be investigated.

## Materials and methods

### Cell culture and low oxygen treatments

The AC16 human cardiomyocyte cell line was purchased from EMD Millipore and the human renal epithelial derived 293T cells were purchased from American Type Culture Collection (Bethesda, MD, U.S.A.). Cells were cultured in Dulbecco’s modified Eagle’s medium (DMEM) containing 12% FBS, and 1% antibiotics (streptomycin and penicillin). AC16 cells were incubated at 37°C in a humid atmosphere with 5% CO_2_ and 95% air. Cells were placed in an Invivo200 cultivator (Ruskin Technology Ltd, U.K.) containing a gaseous mixture of 94% N_2_, 5% CO_2_, and 1% O_2_ at 37°C for durations of 8, 16, 24, 48, and 72 h, respectively. Cells in normoxia group were incubated under the same conditions.

### MiRNA, siRNA, and plasmid DNA transfection

The *miR-138* mimics, *miR-138* inhibitor, and negative control miRNAs were purchased from manufacturer GenePharma (Shanghai, China). AC16 cells were seeded in a six-well plate at 2 × 10^5^/well overnight. Then, the cells were transfected with miRNA mimics, inhibitor, or miR-NC (50 nM) using Lipofectamine RNAiMAX transfection reagent (Invitrogen, U.S.A.) in accordance with the manufacturer’s instructions. Glut1 siRNA, pyruvate dehydrogenase kinase 1 (PDK1) siRNA, or control siRNA was transfected at 100 nM. Overexpression vector of PDK1 or control vector was transfected at 4 µg. Seventy-two hours after transfection, cells were collected for following assays.

### Real-time Reverse Transcription Polymerase Chain Reaction (qRT-PCR)

Total RNA was extracted from AC16 cells using TRIzol® reagent (Invitrogen, U.S.A.) according to the manufacturer’s instructions. The isolated RNA was treated with DNase I (Invitrogen, U.S.A.) to eliminate genomic DNA. cDNA was synthesized using the TaqMan Advanced miRNA cDNA Synthesis Kit (Thermo Fisher Scientific. Inc, U.S.A.). cDNA product was used for the amplification procedure in a 20-µl reaction mixture containing 10-µl SYBR Green PCR Master Mix (Invitrogen, U.S.A.), 7 µl diethylpyrocarbonate-H_2_O, and 1 µl *miR-138* primer and universal primer. The amplification was conducted using the 7900HT Fast Real-Time PCR system (Applied Biosystems, U.S.A.). The protocol consisted of an initial denaturation and enzyme activation at 95°C for 10 min, followed by 35 cycles of 30 s denaturation at 95°C, an attachment of primers for 1 min at 60°C, and extension at 72°C for 30 s, and finally one cycle at 72°C for 10 min for final elongation. U6 was used as an internal control for *miR-138* expression. Relative expression levels of *miR-138* were calculated using the 2^−ΔΔ*C*^_t_ method.

### Luciferase assay

The luciferase assay was performed according to the previous report [19]. The 3′-UTR of PDK1 harboring either the wild-type *miR-138*-binding site or a mutant *miR-138*-binding site was cloned into the psiCHECK-2 vector (Promega, U.S.A.) immediately downstream of the stop codon of the luciferase gene to generate the psiCHECK-PDK1-3′-UTR luciferase reporter plasmid. Plasmid DNA and *miR-138* mimics or control miRNAs were cotransfected into 293T or AC16 cells using Lipofectamine 2000 (Invitrogen Inc., U.S.A.) for 72 h. Luciferase activities were measured with a Dual-Glo Luciferase Assay System (Promega, U.S.A.). Firefly luciferase activity was normalized to *Renilla* luciferase activity. All experiments were performed in triplicate.

### Cell survival

Cell survival rate was determined by MTT (Sigma, U.S.A.) colorimetric assay according to the previous report [19]. Briefly, AC16 cells were seeded in 96-well tissue culture plates at 2 × 10^4^ cells per well and incubated in hypoxic or normal conditions for various hours. Cells were washed with PBS and then incubated in 100 ml of 5 mg/ml MTT solution (Invitrogen Inc., U.S.A.) for 3 h. After incubation, DMSO (Invitrogen Inc., U.S.A.), was added into each well. MTT was converted into purple-colored formazan in living cells and absorbance of solution was taken at 450 nm using the microplate reader Thermo Plate (Rayto Life and Analytical Science Co. Ltd, Germany). All the experiments were performed in triplicate.

### Measurements of glycolysis rate

The glucose uptake assay was performed using the Glucose Uptake Colorimetric Assay Kit (#K676) from BioVision (Milpitas, CA, U.S.A.) according to the manufacturer’s instructions. The lactate production was analyzed by the Lactate Colorimetric/Fluorometric Assay Kit (#K607) from BioVision (Milpitas, CA, U.S.A.) according to the manufacturer’s instructions. The extracellular acidification rate (ECAR) and oxygen consumption rate were measured using the Seahorse XFp Extracellular Flux Analyzer from Agilent (Santa Clara, CA, U.S.A.). Results were repeated three times and normalized by protein concentrations of each test.

### Measurements of intracellular ATP

The intracellular ATP assay was performed using the ATP Assay Kit (colorimetric/fluorometric) from Abcam (#ab83355, Cambridge, U.K.) according to the manufacturer’s instructions. Results were repeated three times and normalized by protein concentrations of each test.

### Measurements of mitochondrial respiration chain activity

The mitochondrial respiration chain activity (complexes I, II, III, IV, and V) was measured using the MitoTox™ Complete OXPHOS Activity Assay Kit (5 Assays) (ab110419) from Abcam, Inc. (Cambridge, MA, U.S.A.) according to the manufacturer’s instructions. Results were repeated three times and normalized by protein concentrations of each test.

### Western blot

Rabbit monoclonal anti-Glut1 (#12939), rabbit monoclonal anti-LDHA (#3582), rabbit monoclonal anti-PDK1 (#13037), and mouse monoclonal anti-β-actin (#3700) were purchased from Cell Signaling Technology (Danvers, MA, U.S.A.). Proteins from cells were extracted using RIPA lysis buffer (Beyotime, China) and separated by SDS/PAGE (10% gel). Subsequently, proteins from gel were transferred on to PVDF membranes. After blocking by 5% nonfat milk for 1 h at room temperature, membranes were probed with primary antibodies at 4°C overnight, followed by incubation with horseradish peroxidase conjugated secondary antibodies (Beyotime, China). After washing completely, membranes were detected by ECL method (Beyotime, China).

### Statistical analysis

Data are expressed as mean ± S.D. Statistical differences amongst different groups were assessed by Student’s *t* test using Prism version 5.0 (GraphPad Software, Inc., La Jolla, CA, U.S.A.). *P*<0.05 was considered to be of statistical significance.

## Results

### Expressions of *miR-138* in cardiac cells are modulated by hypoxia

**Figure 1:**
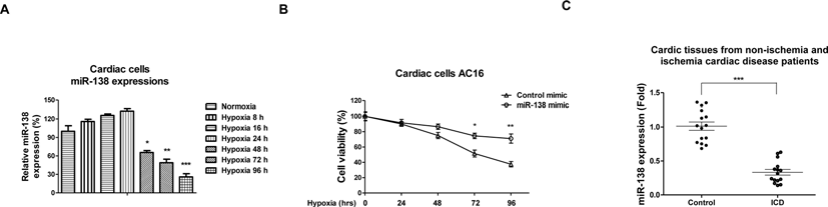
*MiR-138* is down-regulated by long-time hypoxia and negatively correlated with ICD (**A**) Human cardiac cells AC16 were treated with normoxia or hypoxia (1% oxygen) for 8, 16, 24, 48, 72, or 96 h, followed by the measurements of *miR-138* expressions by qRT-PCR. (**B**) AC16 cells were transfected with control mimic or *miR-138* mimic at 50 nM for 48 h, cells were exposed to hypoxia at 0, 24, 48, 72, and 96 h. The cell viability was measured by MTT assay. (**C**) Expressions of *miR-138* were analyzed in cardiac tissues from 15 nonischemia and 15 ICD patients by qRT-PCR. All experiments were performed in triplicate. Data are presented with the indication of mean ± S.D. *: *P*<0.05; **: *P*<0.01; ***: *P*<0.001.

We investigated the roles of *miR-138* in cardiac cells under hypoxia. First, we detected expressions of several miRNAs under hypoxia in human cardiac cell line AC16, of which *miR-138* was one of the most sensitive miRNAs to hypoxia (results not shown). Consistently, qRT-PCR analysis revealed that *miR-138* was slightly induced under hypoxic conditions (1% oxygen concentration) at 8, 16, and 24 h (Figure 1A). Moreover, at 48, 72, and 96 h, we detected that *miR-138* was significantly suppressed under hypoxia (Figure 1A), suggesting that *miR-138* may be involved in the cellular adaptation processes in response to oxygen supply. Expression of *miR-138* was found to be down-regulated in a time-dependent manner and decreased by 5.2-fold at 72-h exposure to hypoxia (Figure 1A). To analyze the potential roles of *miR-138* in cardiac cells, we overexpressed *miR-138* in AC16 cells by transfection with *miR-138* mimics or control miRNAs (Supplementary Figure S1). As we expected, overexpression of *miR-138* contributed to the AC16 cell survival under hypoxic condition at 72 and 96 h (Figure 1B). To test whether *miR-138* changes in cardiomyocytes during ischemia *in vivo*, we analyzed the expressions of *miR-138* from 15 noncardiac ischemia patients and 15 ischemia cardiac disease (ICD) patients. Consistent with *in vitro* results, we found expressions of *miR-138* were significantly decreased in cardiac tissues of ICD patients (Figure 1C), suggesting that *miR-138* plays important functions in human ICD. Taken together, these results demonstrated that *miR-138* protects cardiac cells after long hypoxic exposure time.

### *MiR-138* inhibits glycolysis and promotes mitochondrial respiration

**Figure 2:**
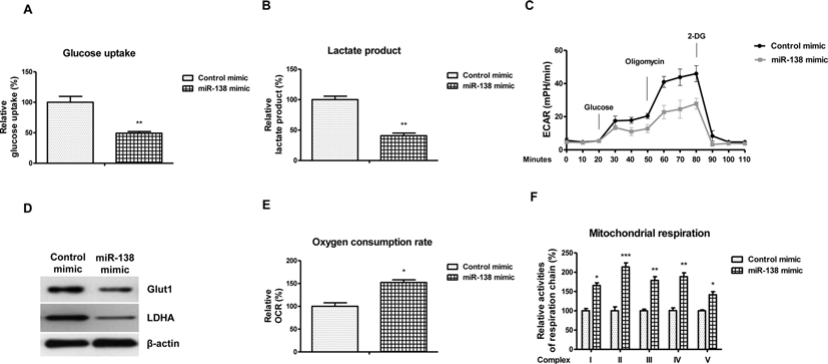
*MiR-138* suppresses glycolysis and promotes mitochondrial respiration of cardiac cells AC16 cells were transfected with control mimic or *miR-138* mimic at 50 nM for 48 h, then (**A**) glucose uptake, (**B**) lactate product, and (**C**) ECAR were analyzed. (**D**) Western blot analysis of Glut-1, LDHA protein expression in AC16 cells with control mimic or *miR-138* mimic transfection. A representative image is presented using β-actin as the loading reference. (**E**) AC16 cells were transfected with control mimic or *miR-138* mimic at 50 nM for 48 h, then the oxygen consumption rate and (**F**) activities of complexes I, II, III, IV, and V from mitochondrial respiration chain were analyzed. All experiments were performed in triplicate. Data are presented with the indication of mean ± S.D. *: *P*<0.05; **: *P*<0.01; ***: *P*<0.001.

Previous studies demonstrated the cellular glycolysis and mitochondrial respiration were regulated by hypoxia, contributing to cell survival with low oxygen supply [10,11]. To explore the potential functions of *miR-138* in the cellular metabolism, we measured the glucose metabolism of AC16 cells under normoxia or hypoxia. We observed that the glucose uptake (Figure 2A), lactate product (Figure 2B), and ECAR (Figure 2C) were significantly inhibited by *miR-138* overexpression. Moreover, glycolysis key enzymes, Glut1 and LDHA were found to be suppressed by *miR-138* (Figure 2D), suggesting that *miR-138* targets the hypoxia-modulated cellular glycolysis in AC16 cells. According to previous report, cells experienced metabolic switch from mitochondrial respiration to glycolysis under hypoxia, we therefore hypothesized that the mitochondrial respiration of human cardiac cells was regulated by *miR-138* [10,11]. the oxygen consumption of AC16 cells was significantly decreased with the increase of *miR-138* (Figure 2E). Consistently, we detected the activities of complexes I, II, III, IV, and V from mitochondrial respiration chain that were significantly decreased by overexpressing *miR-138* (Figure 2F). Taken together, these results suggest that *miR-138* inhibits glycolysis but promotes mitochondrial respiration in human cardiac cells.

### PDK1 is a direct target of *miR-138*

**Figure 3:**
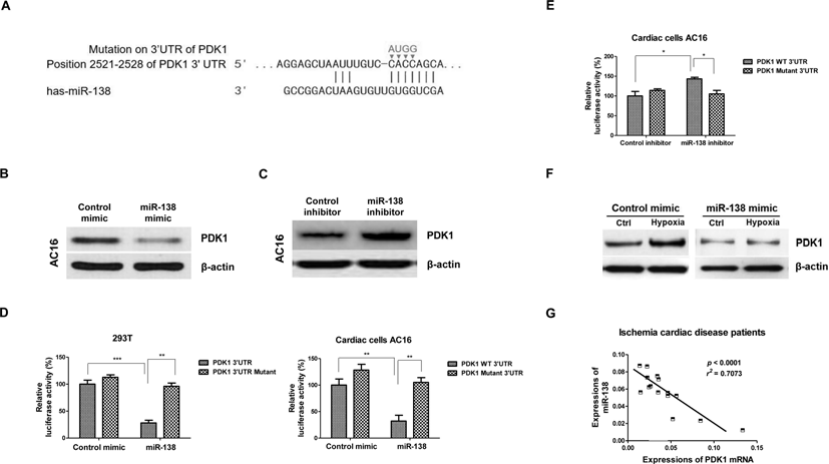
*MiR-138* directly targets PDK1 (**A**) Target prediction from TargetScan.org, microrna.org, and Exiqon. The 3′-UTR region of PDK1 contains binding sites for *miR-138*. (**B**) AC16 cells were transfected with control mimic or *miR-138* mimic at 50 nM for 48 h, Western blot analysis of PDK1 protein expression was performed. (**C**) AC16 cells were transfected with control inhibitor or *miR-138* inhibitor at 50 nM for 48 h, Western blot analysis of PDK1 protein expression was performed. A representative image is presented using β-actin as the loading reference. (**D**) Luciferase assay demonstrated *miR-138* mimic bond to 3′-UTR of PDK1 to attenuate luciferase activity but did not affect the luciferase activity of mutant 3′-UTR of PDK1 in 293T (left) and AC16 (right) cells. (**E**) Luciferase assay demonstrated inhibition of *miR-138* increased luciferase activity of the vector containing wild-type 3′-UTR of PDK1 but did not affect the luciferase activity of mutant 3′-UTR of PDK1 in AC16 cells. (**F**) AC16 cells were transfected with control mimic or *miR-138* mimic for 48 h, cells were treated with or without hypoxia. Western blot analysis of PDK1 protein expression was performed. (**G**) Reverse correlation between *miR-138* expressions and *PDK1* mRNA was analyzed in cardiac tissues from ICD patients. All experiments were performed in triplicate. Data are presented with the indication of mean ± S.D. **: *P*<0.01; ***: *P*<0.001.

To elucidate the underlying molecular mechanisms for the metabolic switch driven by *miR-138*, we performed a bioinformatics analysis using three softwares: TargetScan, microrna.org, and Exiqon to predict the putative *miR-138* target genes. We observed that the PDK1 contained a conservative *miR-13*-binding site in its 3′-UTR predicted by all three softwares (Figure 3A and Supplementary Figure S2). Moreover, the *miR-138*-binding sites on 3′-UTR of PDK1 is conserved in multiple species (Supplementary Figures S3 and S4). PDK1 is a mitochondrial enzyme which inhibits the activity of pyruvate dehydrogenase, a complex of enzymes that converts cytosolic pyruvate into mitochondrial acetyl-CoA [11]. It has been known that PDK1 plays important roles in maintaining cellular glucose metabolism [11]. In addition, inhibition of PDK1 with either siRNAs or inhibitor shifts the metabolism of cancer cells from glycolysis to mitochondrial respiration [20]. Given that *miR-138* inhibited glycolysis and promoted mitochondrial respiration (Figure 2), it was strongly supported that PDK1 may be targetted by *miR-138*. To confirm this prediction, we transfected *miR-138* mimics or control mimics into AC16 cells, then measured the protein expression of PDK1. Our results from Western blot showed overexpression of *miR-138* suppressed PDK1 expression in AC16 cells (Figure 3B). Moreover, AC16 cells with transfection of *miR-138* inhibitor showed up-regulated PDK1 expression compared with control inhibitor transfection (Supplementary Figure 5 and Figure 3C). To test whether PDK1 is a direct target of *miR-138*, we cloned the wild-type or binding site mutant 3′-UTR of PDK1 (Figure 3A) into pmiR-report vector and cotransfected *miR-138* mimics or negative control with vectors in 293T and AC16 cells. The luciferase activity was significantly reduced in *miR-138* and wild-type 3′-UTR cotransfection cells compared with that of the negative control and wild-type 3′-UTR cotransfection (Figure 3D). Importantly, the luciferase activity was not reduced in *miR-138* and binding site mutant 3′-UTR cotransfection cells (Figure 3D). Consistently, luciferase activity was significantly increased in *miR-138* inhibitor and wild-type 3′-UTR cotransfection cells compared with that of the control inhibitor and wild-type 3′-UTR cotransfection (Figure 3E). To investigate whether *miR-138* could target PDK1 under hypoxia, we treated AC16 cells without or with *miR-138* overexpression with hypoxia. Results in Figure 3F demonstrated that under hypoxia, PDK1 expression was induced. AC16 cells with *miR-138* transfection significantly suppressed PDK1 expression under both normoxia and hypoxia conditions (Figure 3F). In addition, we observed a significant negative correlation between *PDK1* mRNA expressions and endogenous *miR-138* levels in ICD patient samples, indicating that *miR-138* could target PDK1 *in vivo* (Figure 3G). All these findings suggested that *miR-138* inhibited PDK1 protein expression by direct binding to its 3′-UTR.

### Hypoxia suppresses cardiac cells’ viability through increased glycolysis and decreased mitochondrial respiration

**Figure 4:**
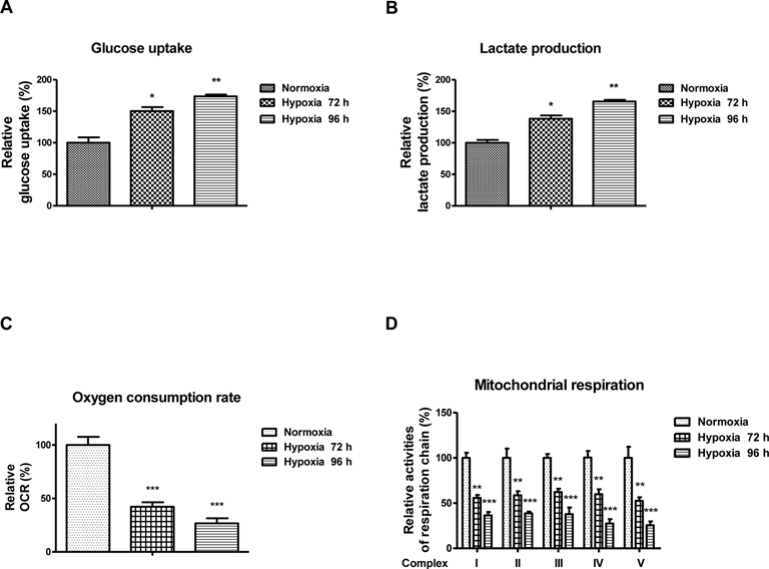
Hypoxia induces glycolysis and suppresses mitochondrial respiration AC16 cells were treated with normoxia, hypoxia at 72 or 96 h. The (**A**) glucose uptake, (**B**) lactate product, (**C**) oxygen consumption rate, and (**D**) mitochondrial respiration were analyzed. All experiments were performed in triplicate. Data are presented with the indication of mean ± S.D. *: *P*<0.05; **: *P*<0.01; ***: *P*<0.001.

**Figure 5:**
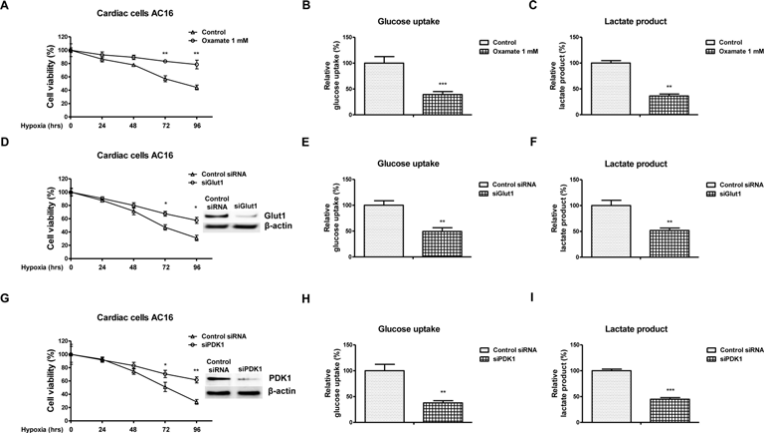
Inhibition of glycolysis contributes to cardiac cells’ survival under hypoxia (**A**) AC16 cells were treated with or without oxamate at 1 mM for 48 h. Cells were exposed to hypoxia at 0, 24, 48, 72, and 96 h. Cell viability was analyzed by MTT assay, (**B**) glucose uptake and (**C**) lactate product were measured. (**D**) AC16 cells were transfected with control siRNA or siGlut1 for 48 h. Cells were exposed to hypoxia at 0, 24, 48, 72, and 96 h, followed by measurement of cell viability by MTT assay, (**E**) glucose uptake, and (**F**) lactate product were measured. The expression of Glut1 was analyzed by Western blot. (**G**) AC16 cells were transfected with control siRNA or siPDK1 for 48 h. Cells were exposed to hypoxia at 0, 24, 48, 72, and 96 h, followed by measurement of cell viability by MTT assay, (**H**) glucose uptake and (**I**) lactate product were measured. The expression of PDK1 was analyzed by Western blot. All experiments were performed in triplicate. Data are presented with the indication of mean ± S.D. *: *P*<0.05; **: *P*<0.01. ***: *P*<0.001.

The above results demonstrated roles of *miR-138* in regulating cellular metabolism under hypoxia, we next assessed whether the hypoxia-induced *miR-138* led to decreased viability of cardiac cells through regulation of glucose metabolism. Consistent with previous reports, cells exhibited up-regulation of glycolysis rate and decreased mitochondria respiration under hypoxia at 72 and 96 h (Figure 4A–D), indicating inhibition of glycolysis might protect cardiac cells from hypoxia-induced dysfunction [7]. To test this, we treated AC16 cells with glycolysis inhibitor, oxamate under normoxia and hypoxia. Cardiac cells displayed attenuated cell viability and glycolysis rate with glycolysis inhibitor treatments under hypoxia (Figure 5A–C). Moreover, we observed similar results with knockdown Glut1 or PDK1 by siRNA. AC16 cells showed decreased cell viability and glycolysis rate with knocking down of Glut1 or PDK1 (Figure 5D–I). Taken together, these results demonstrated that inhibition of glucose metabolism under hypoxia could protect cardiac cells.

### Overexpression of *miR-138* increases the cardiac cells’ viability through targetting PDK1 under hypoxia

**Figure 6:**
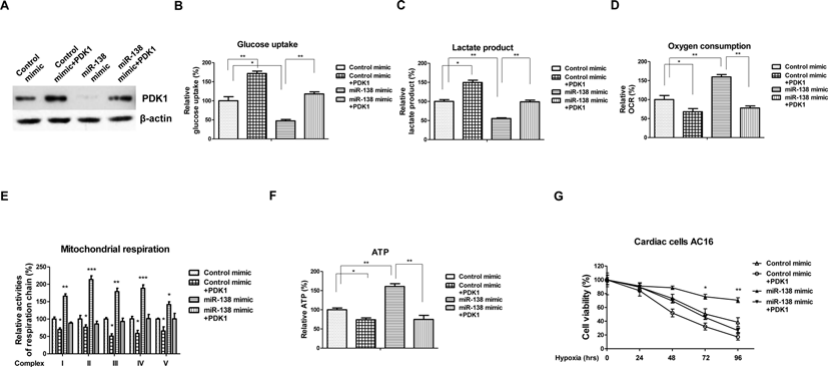
Overexpression of *miR-138* protects cardiac cells against hypoxia through targetting PDK1 (**A**) AC16 cells were transfected with control mimic, control mimic plus PDK1, *miR-138* mimic alone, or cotransfection of *miR-138* and PDK1 for 48 h. Western blot analysis of PDK1 protein expression. A representative image is presented using β-actin as the loading reference. (**B**) AC16 cells were transfected with control mimic, control mimic plus PDK1, *miR-138* mimic alone, or cotransfection of *miR-138* mimic and PDK1 overexpression plasmid for 48 h. The glucose uptake, (**C**) lactate product, (**D**) oxygen consumption, (**E**) mitochondrial respiration, and (**F**) intracellular ATP were measured. (**G**) AC16 cells were transfected with control mimic, control mimic plus PDK1, *miR-138* mimic alone, or cotransfection of *miR-138* and PDK1 for 48 h. Cells were exposed to hypoxia at 0, 24, 48, 72, and 96 h, followed by the detection of cell viability by MTT assay. All experiments were performed in triplicate. Data are presented with the indication of mean ± S.D. *: *P*<0.05; **: *P*<0.01; ***: *P*<0.001.

Our results demonstrate overexpression of *miR-138* contributed to the AC16 cell survival under hypoxia (Figure 1C). To verify whether the *miR-138*-mediated anti-apoptotic effects under hypoxia were through the modulation of glucose metabolism, we overexpressed PDK1 in *miR-138* overexpressing AC16 cells by cotransfection of control vector or PDK1 overexpression vector with *miR-138* (Figure 6A). Western blot results in Figure 6A demonstrated transfection of PKD1 into *miR-138* overexpressing cells could efficiently restore the original protein levels of PDK1. Overexpression of *miR-138* promoted glucose uptake and lactate product (Figure 6B,C), decreased oxygen consumption, mitochondrial complex activity, and intracellular ATP (Figure 6D–F). Restoration of PDK1 recovered glucose uptake (Figure 6B) and lactate product (Figure 6C) in AC16 cells. Consistently, the oxygen consumption rate (Figure 6D), mitochondrial respiration (Figure 6E), and intracellular ATP (Figure 6F) were decreased by PDK1 restoration in *miR-138* overexpressing cells. We next assessed the cell viability of AC16 cells with or without PDK1 restoration under hypoxia. As we expected, restoration of PDK1 in *miR-138* overexpressing cells resensitized cells to low oxygen (Figure 6G), indicating that the *miR-138*-mediated anti-apoptotic effects under hypoxia was through directly targetting PDK1.

## Discussion

During cardiac surgery, the heart is continually exposed to ischemia and myocardial infarction due to lack of oxygen supply from blood flow. These ischemic episodes cause apoptosis of cardiomyocytes, resulting in dysfunction of heart. To prevent the expansion of these apoptotic regions, the *in vitro* hypoxic cardiac cells model contributes to understand the potential mechanisms of the hypoxia-induced cardiomyocytes dysfunction. In the present study, we investigated the roles of *miR-138* during low oxygen exposure of cardiac cells. qRT-PCR analysis revealed that *miR-138* was slightly induced under hypoxic conditions at early time point, suggesting *miR-138* may be involved in the cellular adaptation processes in response to oxygen supply. Moreover, *miR-138* was significantly suppressed by hypoxia at 48, 72, and 96 h, suggesting that targetting *miR-138* might contribute to the prevention of the myocardial infarction mediated heart failure because hypoxia may persist within the infarct for days or weeks, exacerbating the injury. Recent studies demonstrated similar functions of *miR-138* in pulmonary artery smooth muscle cells (PASMCs) during hypoxia as our observations [21]. They reported expression of exogenous *miR-138* suppressed the PASMC apoptosis and prevented caspase activation through targetting serine/threonine kinase (Mst1).

Previous study demonstrated that overexpression of *miR-138* significantly benefited cardiomyocytes from hypoxia-induced cell apoptosis [19]. They indicated that the hypoxia could induce the cardiomyocyte apoptosis, which is the main reason for the cardiac diseases. In the present study, we established the cardiac cell death model under hypoxia. Under conditions of hypoxia, oxygen and nutrients supply are severely hampered, leading to energy deprivation. In response to hypoxia, the ischemic myocardium switches from respiration to glycolytic energy metabolism, with increased glucose consumption, lactic acid production, and lower intracellular pH [22]. Our results showed that overexpression of *miR-138* significantly suppressed PDK1 expression through directly targetting 3′-UTR of *PDK1* mRNA, resulting in a metabolic switch from the hypoxia-induced glycolysis to mitochondrial respiration. This metabolic switch contributes to protect cardiac cells from hypoxia, presenting local delivery of *miR-138* as an approach against cardiac cells dysfunction during surgery or ischemia.

In conclusion, we illustrated that overexpression of *miR-138* inhibits the hypoxia-induced cardiac cells death via suppression of the dysregulated glycolysis and recovery of mitochondrial respiration. We report that *miR-138* directly targets PDK1, an enzyme which promotes glycolysis. The present study leads to intervention of novel therapeutic strategies against cardiac cells dysfunction during surgery or ischemia.

## Supporting information

**Figure 1:** 

**Figure 2:** 

**Figure 3:** 

**Figure 4:** 

**Figure 5:** 

## Abbreviations

ECAR: extracellular acidification rate
Glut1: Glucose transportor 1
ICD: ischemia cardiac disease
PASMC: pulmonary artery smooth muscle cells
PDC: pyruvate dehydrogenase complex
PDK1: pyruvate dehydrogenase kinase 1
qRT-PCR: Real-time Reverse Transcription Polymerase Chain Reaction
