# Supplementary material for: MiR-138 protects cardiac cells against hypoxia through modulation of glucose metabolism by targetting pyruvate dehydrogenase kinase 1
Source: Biosci Rep. 2017 Nov 21;37(6):BSR20170296. doi: 10.1042/BSR20170296 (PMC5696451; doi:10.1042/BSR20170296)

Supplementary data

Figure 1

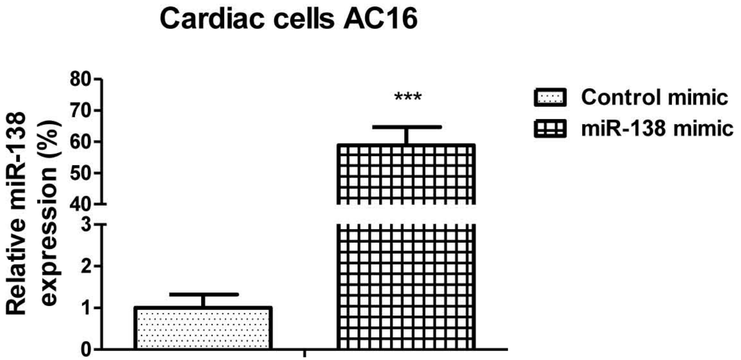

Supplementary data

Figure 2

Targetscan

	Predicted consequential pairing of target region (top) and miRNA (bottom)	Site type
Position 2521-2528 of PDK1 3' UTR<br>hsa-miR-138-5p	5' ...AGGAGCUAAUUUGUC-CACCAGCA...<br>||| ||||||| 3' GCCGGACUAAGUGUUGUGGUCGA	8mer

microRNA.org

hsa-miR-138/PDK1 Alignment

```
     3' gccgGACUAAGUGUUGUGGUCGa 5' hsa-miR-138
            ||  |||:::   |||||||
2507:5' ggagCUAAUUUGU-CCACCAGCa 3' PDK1
```

mirSVR score:	-0.7625
PhastCons score:	0.6093

Exiqon

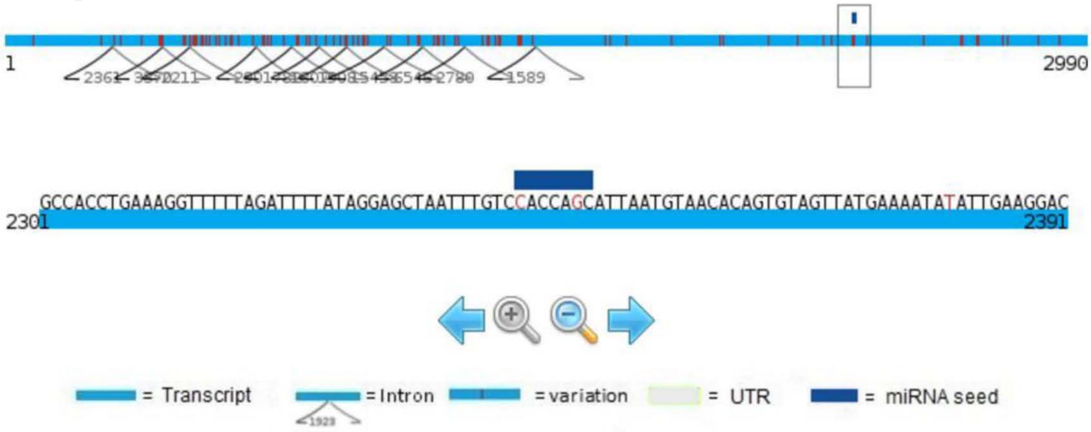

Visual representation of hsa-miR-138-5p on PDK1

For each transcript, the interaction is shown as a vertical blue line above the sequence. It is possible to zoom to sequence level for each transcript by clicking Details

ENST00000410055

Supplementary data

Figure 3

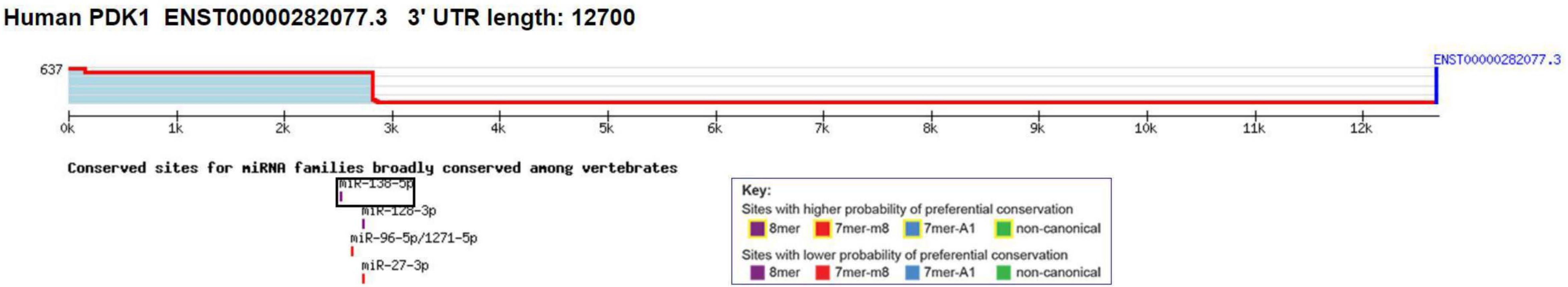

Figure 4

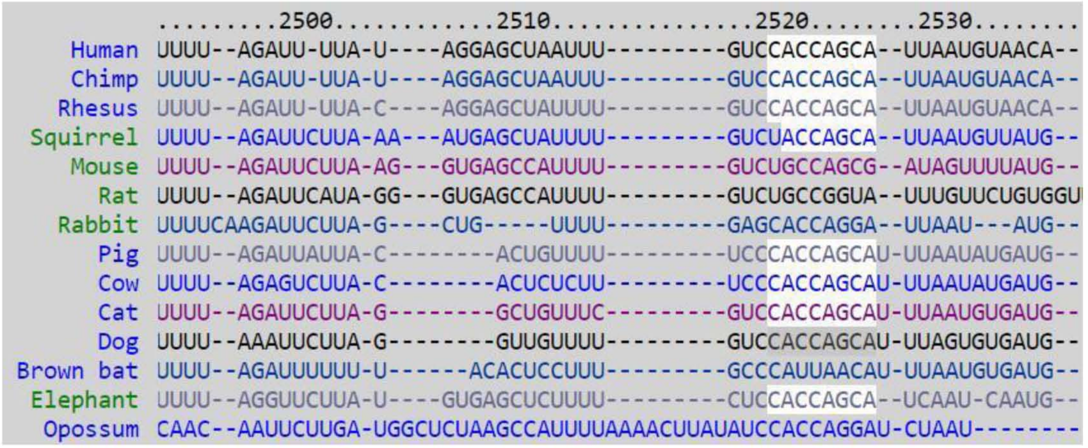

Figure 5

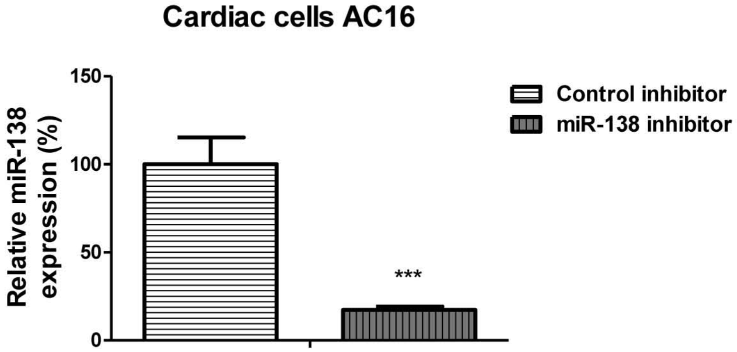

Supplement: Supplementary file 1 [file bsr20170296_Supp1.pdf]
